# *Enterobacteriaceae* in the Human Gut: Dynamics and Ecological Roles in Health and Disease

**DOI:** 10.3390/biology13030142

**Published:** 2024-02-23

**Authors:** Maria Ines Moreira de Gouveia, Annick Bernalier-Donadille, Gregory Jubelin

**Affiliations:** Université Clermont Auvergne, INRAE, MEDIS UMR454, F-63000 Clermont-Ferrand, France; maria.inesmgouveia@gmail.com (M.I.M.d.G.); annick.bernalier@inrae.fr (A.B.-D.)

**Keywords:** human gut microbiota, *Enterobacteriaceae*, *Escherichia coli*, gut dysbiosis

## Abstract

**Simple Summary:**

The human gastrointestinal tract harbors a complex and dynamic population of microorganisms (mostly bacteria and viruses) that are called the gut microbiota. This microbial community is known to exert a marked influence on the host health. Among the different bacterial families present in the gut microbiota, *Enterobacteriaceae* represents only a small fraction of total bacteria in healthy individuals, whereas it can abnormally proliferate in the gut of individuals affected by intestinal or extra-intestinal diseases such as obesity, inflammatory bowel disease or metabolic disorders. Our review explores the recent knowledge discussing the dynamics of *Enterobacteriaceae* in the human gut in healthy or disease-associated conditions. We also describe emerging mechanistic studies that could explain how *Enterobacteriaceae* proliferate in cases of gut microbiota imbalance in patients as well as the consequences such expansions could have on host health.

**Abstract:**

The human gut microbiota plays a crucial role in maintaining host health. Our review explores the prevalence and dynamics of *Enterobacteriaceae*, a bacterial family within the Proteobacteria phylum, in the human gut which represents a small fraction of the gut microbiota in healthy conditions. Even though their roles are not yet fully understood, *Enterobacteriaceae* and especially *Escherichia coli* (*E. coli*) play a part in creating an anaerobic environment, producing vitamins and protecting against pathogenic infections. The composition and residency of *E. coli* strains in the gut fluctuate among individuals and is influenced by many factors such as geography, diet and health. Dysbiosis, characterized by alterations in the microbial composition of the gut microbiota, is associated with various diseases, including obesity, inflammatory bowel diseases and metabolic disorders. A consistent pattern in dysbiosis is the expansion of Proteobacteria, particularly *Enterobacteriaceae*, which has been proposed as a potential marker for intestinal and extra-intestinal inflammatory diseases. Here we develop the potential mechanisms contributing to *Enterobacteriaceae* proliferation during dysbiosis, including changes in oxygen levels, alterations in mucosal substrates and dietary factors. Better knowledge of these mechanisms is important for developing strategies to restore a balanced gut microbiota and reduce the negative consequences of the *Enterobacteriaceae* bloom.

## 1. Proteobacteria/*Enterobacteriaceae* in the Healthy Human Gut

### 1.1. Taxonomy, Diversity and Abundance

The human gut microbiota harbors approximately 10^14^ microorganisms, comprising more than 1000 bacterial species belonging to only few bacterial phyla [1]. Firmicutes and Bacteroidota, comprising 90% of the overall phylum composition, stand out as the predominant microbial phyla in the gut. Actinobacteria, Proteobacteria, Fusobacteria and Verrucomicrobia are also found as part of the normal gut microbiota but are represented to a lesser extent [2]. The phylum Proteobacteria is systematically present in the gut of healthy humans with a low abundance (less than 5% of total microbial abundance) [3]. Based on 16S rDNA sequence analysis, the Proteobacteria phylum is divided into six classes: *Alphaproteobacteria*, *Betaproteobacteria*, *Gammaproteobacteria*, *Deltaproteobacteria*, *Epsilonproteobacteria* and *Zetaproteobacteria*. In the human gut, *Gammaproteobacteria* and *Deltaproteobacteria* are the most commonly found, *Gammaproteobacteria* being mainly represented by the *Enterobacteriaceae* family. The *Enterobacteriaceae* family comprises both commensals and opportunistic disease-causing pathogens. However, *Enterobacteriaceae* usually constitutes less than 1% of the healthy gut microbiota. This family includes a diversity of bacterial genera including *Escherichia*, *Enterobacter* and *Klebsiella*. The species *Escherichia coli* was shown to be, by far, the most dominant *Enterobacteriaceae* in healthy humans [4]. For these reasons, most data available in the literature regarding *Enterobacteriaceae* in the gut microbiota frequently concern *E. coli*, even if other minor bacterial species could have important functions as well.

Cross-sectional studies of human adults demonstrated that *E. coli* is a member of the intestinal microbiome of over 90% of individuals [5]. The number of distinct *E. coli* strains per host was also assessed in individuals and the average number was found to be between 1.1 and 3.5 *E. coli* strains per individual [6]. Based on molecular analysis, the different *E. coli* strains can be divided into nine defined phylogenetic groups (A, B1, B2, C, D, E, F, G and H). The prevalence of *E. coli* phylogenetic groups in the gut of healthy individuals varies between studies and appears to depend on different factors such as geographic location and diet. While phylogroup A was dominant in industrialized countries in the 1980s [5], the proportion of phylogroup B2 strongly increased over the last 30 years and is now as prevalent as phylogroup A in healthy individuals [7]. Also, the presence of a dominant strain from phylogroups E and F appears to tolerate the co-occurrence of other minor *E. coli* strains, whereas the establishment of a dominant strain from phylogroup B2 seems to limit gut colonization by other *E. coli* populations [8].

### 1.2. Strain Residency in the Gut

Longitudinal studies in healthy individuals have reported that the gut microbiota composition is relatively stable at high taxonomic levels. Nevertheless, recent studies present evidence suggesting a significant turnover rate at the strain level, at least within populations of *Enterobacteriaceae* [4,9,10]. In one of these studies, strain residency was evaluated in eight participants for an average time of 500 days [4]. Only 31% of *Enterobacteriaceae* clones were considered as long-term residential strains, whereas the others were defined as short-term transient strains. It is crucial to emphasize that the permanence of strains can vary significantly among individuals. While some individuals may host several resident strains for extended periods, others may experience the successive emergence and disappearance of clonal populations over time [4]. Thus, it appears that *Enterobacteriaceae* residency is a dynamic process and resident clones turn over at different rates in individuals. *E. coli* strains are not all equal with respect to their ability to colonize and reside in the adult human gut. Depending on their properties, ingested *E. coli* strains are able to establish in the gut and become resident for a long period or are quickly lost at the rate of gut transit. Several factors are thought to influence *E. coli* residency in the gut such as socio-economic factors, diet, health, travel or exposure to antibiotics. However, the rules governing *E. coli* residency in the gut are far from being fully understood. Nonetheless, it has been shown that long-lived resident *E. coli* are phylogenetically distinct from short-lived transients and belong preferentially to phylogenetic groups A, B2 and F [4]. Gene association studies are now warranted to better define residency-associated traits and understand this important aspect of microbial ecology.

### 1.3. Functional Roles of Enterobacteriaceae in the Human Gut

Despite the fact that *Enterobacteriaceae* are detected in nearly all individuals, the ecological roles of this bacterial family in the human gut are poorly understood. As of now, the functions assigned to *Enterobacteriaceae* include the following:

- Maintenance of an efficient anaerobic environment in the gut

As facultative anaerobes, most *Enterobacteriaceae*, and in particular *E. coli*, deplete oxygen diffusing from the mucosal surface and therefore participate to the establishment of a perfect environment for strict anaerobes which constitute almost all members of the gut microbiota. Several works suggest that *E. coli* develops a relationship with the anaerobic members of the gut microbiota. Within the gastrointestinal tract, anaerobic bacteria break down complex polysaccharides, releasing simple carbohydrates that *E. coli* metabolizes for its growth. In return, *E. coli* helps create an anaerobic environment by scavenging oxygen [11]. This function is important not only in adults but also in the neonates. Indeed, *E. coli* is one of the first bacteria to colonize the gut of neonates at birth. This newly established and rapidly growing *E. coli* population then changes the structure and function of the epithelial cells in ways that appear crucial for healthy microbiome development [12]. In particular, oxygen consumption by *E. coli* and other facultative anaerobes creates hospitable conditions for strict anaerobes to colonize and become dominant inhabitants of the gut [13].

- Production of vitamins

In humans and animals, vitamin K, also referred as menaquinone, has multiple roles and one of them is to be a co-factor for many important enzymes. *E. coli* is known to produce menaquinone during anaerobic growth and uses it as an electron carrier in the respiratory chain [14]. With other members of the gut microbiota, *E. coli* modulates menaquinone concentrations in the human gut and contributes to supplying the host with its vitamin K requirement [15]. *E. coli* is also able to synthesize vitamin B_12_ through modifications of corrinoids [16]. Vitamin B_12_, also known as cyanocobalamin, is atypical among vitamins because it is not produced by plants or the majority of vertebrates. Instead, it is exclusively synthesized by bacteria and archaea. The essential nature of these pathways makes many eukaryotes, including humans, dependent on microbial corrinoids. Nevertheless, the direct impact of gut microbial communities on the levels of cobalamin (vitamin B_12_) in the host is a matter of debate. Indeed, cobalamin produced in the colon, where microbial numbers are highest, is probably not bioavailable for the host because the receptors necessary for absorbing the vitamin are mostly found in the small intestine. In humans, diet is instead more likely to constitute the primary source of cobalamin [17]. Nevertheless, the biological significance of vitamin production by *Enterobacteriaceae* and contribution to host requirement is probably very low because (i) they represent only a small population in the gut microbiota and (ii) vitamin synthesis in the gut relies on multiple bacteria from diverse phyla and results from complex microbial–microbial cooperation [18].

- Protection against gut pathogen infections

The best characterized role of *Enterobacteriaceae* in gut homeostasis concerns its contribution to resistance against colonization by exogenous pathogens. Protection offered by the gut microbiota occurs through different mechanisms, including secretion of antimicrobial products, nutrient competition, support of epithelial barrier integrity and immune activation [19]. In the case of commensal *E. coli*, the protective mechanisms are thought to rely mainly on bacteriocin synthesis and competition for nutrient resources that can limit the growth of enteric pathogens. As an example, a study from Hudault et al. showed that colonization of germfree mice by an *E. coli* strain had a protecting effect against *Salmonella enterica* serovar *Typhimurium* infection [20]. Competition for the same nutritional substrates between commensal and pathogenic *E. coli* strains has been also largely studied in the literature. Commensal *E. coli* consumes monosaccharides such as fucose, mannose, galactose, ribose, arabinose and N-acetyl glucosamine in the mucus layer, which are carbohydrates that are also used by enterohaemorrhagic *E. coli* (EHEC) O157:H7 during infection. Several in vivo mouse studies demonstrated that commensal *E. coli* can prevent EHEC infection due to competition for nutrients such as carbohydrates, amino acids and organic acids [21,22,23,24].

## 2. Proteobacteria/*Enterobacteriaceae* Expansion in Dysbiosis Associated with Human Disease

### 2.1. Proteobacteria Bloom: A Marker of Gut Microbiota in Disease

It has been demonstrated that the gut microbiota contributes to health and the well-being of the individual [25,26]. Any disruption in the composition of the gut microbiota, called dysbiosis, has impacts on host health. Dysbiosis can be defined by typical features such as a decrease in microbial diversity and/or richness, an altered ratio of Bacteroidetes/Firmicutes, a decrease in beneficial species (i.e., *Bifidobacterium*, *Faecalibacterium prauznitsii*…) and/or an increase in deleterious bacteria such as Proteobacteria. Indeed, Proteobacteria usually represent less than 5% of the healthy gut microbiota, but this phylum can reach 10–90% in the gut of patients suffering from various diseases, especially inflammatory ones [27]. Currently, several studies have shown that the composition of the gut microbiota of patients suffering from different diseases differs comparing to the healthy individual. Intestinal dysbiosis has been observed in various diseases such as obesity, allergic disorders, diabetes, autism, colorectal cancer, Alzheimer’s, Parkinson’s or cardiovascular diseases.

Among the multiple taxonomic variations associated with gut dysbiosis, the most consistent and robust ecological pattern is an expansion of facultative anaerobic bacteria belonging to the phylum Proteobacteria. While information at a lower taxonomic level than the Proteobacteria phylum is not systematically described in the different works, the *Enterobacteriaceae* family appears to have a prominent place for explaining the expansion of Proteobacteria associated with disease. From these observations, several groups proposed that an enrichment of Proteobacteria/*Enterobacteriaceae* in the gut is a marker for an unstable microbial community and a potential diagnostic criterion for disease [27,28,29]. Among the works correlating disease state and gut dysbiosis, gut inflammation at low or high levels appears to be associated with expansion of *Enterobacteriaceae* in various disease contexts such as inflammatory bowel diseases, colorectal cancer or metabolic syndrome [30,31,32,33]. Naturally, a legitimate question concerns the causal relationship between *Enterobacteriaceae* bloom and disease state, asking what is the cause and what is the consequence. If the answer is not as simple as the question is, numerous works with mechanistic insights have been performed in the last decade to highlight (i) the conditions that could cause proliferation of *Enterobacteriaceae* in the gut of patients and (ii) the potential consequences of such expansion on health status and disease progression.

### 2.2. Causes of Enterobacteriaceae Proliferation in Dysbiotic Conditions

**Figure 1:**
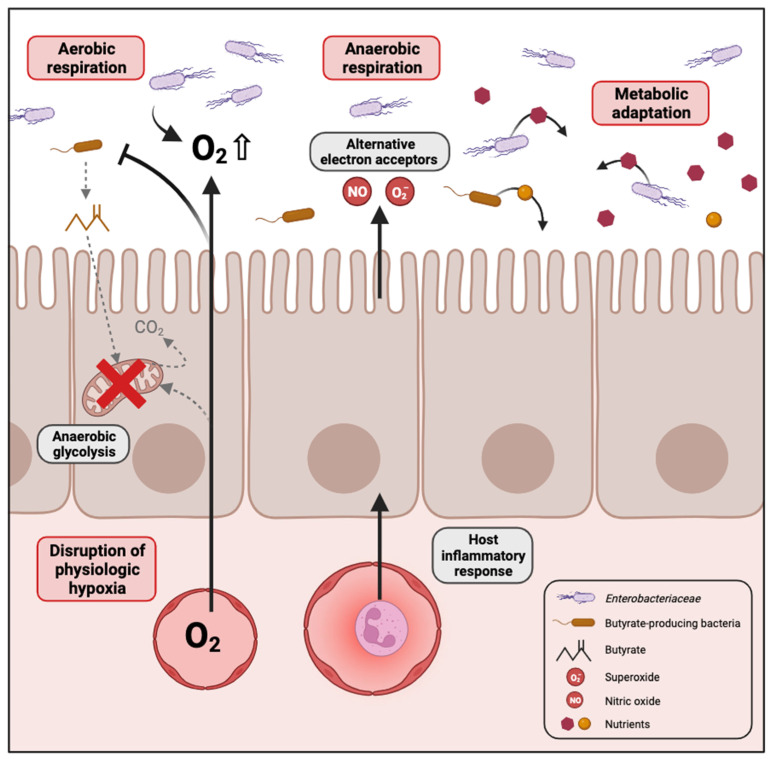
Mechanisms contributing to the expansion of *Enterobacteriaceae* in the dysbiotic gut. In conditions where high levels of oxygen reach the gut lumen, for example when enterocytes switch from mitochondrial fatty acid β-oxidation to anaerobic glycolysis, the facultative anaerobes of *Enterobacteriaceae* proliferate through aerobic respiration. In cases of gut inflammation, an increase in the availability of various electron acceptors promotes anaerobic respiration by *Enterobacteriaceae*. Changes in the gut environment caused by disease-associated dysbiosis can lead to *Enterobacteriaceae* expansion as a result of their adaptability to metabolize potential arising substrates. Created with Biorender.com.

Several mechanisms have been proposed in the literature that could explain the bloom of *Enterobacteriaceae* in the dysbiotic gut, which are summarized in Figure 1.

#### 2.2.1. Disruption of Physiologic Hypoxia

The gastrointestinal tract in humans is hypoxic, characterized by an oxygen gradient extending from the colonic mucosal surface to the gut lumen. This oxygen variation influences the structure of the intestinal microbial community, favouring the establishment of predominantly obligate anaerobes during homeostasis [34,35]. Furthermore, the microbiome plays a key role in sustaining the hypoxic environment within the intestine, which is essential for nutrient absorption, intestinal barrier function and the activation of innate and adaptive immune responses in mucosal cells. Emerging evidence supports that oxygen diffusion within the gut lumen can be a driver of gut dysbiosis [36]. Indeed, an increase in the oxygen level in the gut would destabilize the oxygen-sensitive strict anaerobes and promote the expansion of facultative anaerobes [37]. The majority of Proteobacteria are facultative anaerobes, meaning they have the ability to thrive in the presence of oxygen. This confers a notable competitive edge over beneficial obligate anaerobes in environments where oxygen is present. For instance, antibiotic treatment or inflammation are conditions known to promote *Enterobacteriaceae* expansion. Consistent with this idea, metagenomic analysis in the murine model of colitis identified oxygen respiration as a dominant signature associated with commensal *E. coli* expansion in the gut [38,39].

Butyrate, a short-chain fatty acid, is generated as a metabolite by obligate anaerobes during the breakdown of dietary fiber in the colon. Interestingly, it has been shown that elevated Proteobacteria levels and diminished populations of butyrate-producing bacteria serve as a microbial signature for dysbiosis in various chronic diseases such as inflammatory bowel disease [40], irritable bowel syndrome [41], colorectal cancer [31], diabetes [42] and obesity [43]. For instance, streptomycin treatment leads to a depletion of bacteria belonging to the class Clostridia which are important producers of butyrate. In normal conditions, colonocytes are hypoxic because they consume oxygen through mitochondrial β-oxidation of microbiota-derived butyrate to CO_2_, which represents their main pathway for producing energy [44]. Depletion of butyrate-producing bacteria by antibiotic treatment or other dysbiotic conditions would reduce luminal butyrate levels, resulting in a metabolic reorientation of intestinal epithelial cells towards anaerobic glycolysis, a process that does not consume oxygen [45]. The consequent increase in the amount of oxygen emanating from the colonic surface drives an expansion of facultative anaerobes such as *Enterobacteriaceae* in the gut [46]. Colonocyte metabolism serves as a pivotal regulator for gut microbiota, orchestrating a transition between balanced, homeostatic communities and imbalanced, dysbiotic states.

#### 2.2.2. Inflammation Generates Electron Acceptors for Anaerobic Respiration

Intestinal inflammation also contributes to an increased availability of various electron acceptors that promote anaerobic respiration by *Enterobacteriaceae* [47]. The host inflammatory response produces reactive nitrogen species and reactive oxygen species that, upon diffusion into the gut lumen, will react with organic sulphides, tertiary amines or other molecules to form S-oxides, N-oxides and nitrate. *Enterobacteriaceae* can use these molecules as terminal electron acceptors for anaerobic respiration by expressing DMSO, TMAO and nitrate reductases, respectively [48,49]. For instance, *E. coli* encodes two DMSO reductases (*dmsABC*, *ynfFGH*), three TMAO reductases (*torCAD*, *torYZ*, *yedYZ*) and three nitrate reductases (*narGHJI*, *narZYWV*, *napFDAGHBC*). Such a rich environment for alternative electron acceptors therefore confers a growth advantage for *Enterobacteriaceae* in inflammatory conditions. Indeed, anaerobic respiration is considered a process more efficient for energy production than fermentation made by other members of the gut microbial community.

#### 2.2.3. Metabolic Adaptation to Dysbiotic Conditions

Dysbiosis often causes significant changes in the gut environment and may influence the diversity and concentrations of nutrients available in the lumen that the different members of the gut microbiota can utilize with variable efficiency. *Enterobacteriaceae* and particularly *E. coli* present a dynamic genome structure leading to a high level of genome plasticity. Moreover, *Enterobacteriaceae* have been shown to be the major source of variable genes in the gut microbiome between healthy individuals, whereas they represent only a minor population, indicating that this family brings variability in gut microbial gene function [50]. Consistent with these observations, changes in the gut environment caused by disease-associated dysbiosis can lead to *Enterobacteriaceae* expansion as a result of a better adaptability to metabolize potential arising substrates. Different studies have already explored microbial metabolic adaptation, and some nutrient sources available to *Enterobacteriaceae* during intestinal dysbiosis are summarized in Table 1. Although some of this research has been conducted with enteric pathogens, it is reasonable to believe that most traits can be transposed to commensal *Enterobacteriaceae*.

- Glycerol in cystic fibrosis patients

Cystic fibrosis is a genetic disease affecting the normal functions of multiple organs including the lungs, pancreas and gastrointestinal tract with a pronounced inability of patients to digest and absorb proteins and lipids [61]. A gut dysbiosis notably characterized by a bloom of *Enterobacteriaceae* is associated with cystic fibrosis [62]. Indeed, *E. coli* that normally represents less than 1% of the gut microbiota can reach more than 90% in the fecal microbiota of young children with cystic fibrosis, compared with age-matched healthy controls [63]. In 2018, Matamouros et al. isolated *E. coli* strains from fecal samples of young children suffering from this disease and showed an increased growth rate in the presence of glycerol as a sole carbon source [59]. Glycerol is a major component of fatty acids of intestinal fat, present in high concentrations in the gut of patients as a result of fat malabsorption. In addition, cystic fibrosis and control *E. coli* isolates have differential gene expression when grown in the presence of glycerol, likely resulting in a better growth rate of the patient strains. The authors suggest that *E. coli* expansion in the gut of cystic fibrosis children can be explained through adaptation and/or selection of *E. coli* strains possessing a high ability to metabolize glycerol [59].

- Intestinal mucosa-derived substrates

In the case of diseases marked by an important gut inflammation, alteration of the gut environment can generate a unique set of carbon sources available to *Enterobacteriaceae* to outgrow other members of the gut microbiota. For instance, inflammation causes the shedding of epithelial cells in the lumen and alterations of the mucus layer. Components derived from the epithelial cell membrane such as phosphatidylcholine and phosphatidylethanolamine increase in this context. Ethanolamine (EA) is derived from phosphatidylethanolamine and can be used as a source of carbon and/or nitrogen by a variety of species in the Firmicutes, Actinobacteria and Proteobacteria phyla, as well as by pathogenic species such as *Salmonella* and *Pseudomonas* [64]. Utilization of EA involves breaking ethanolamine into ammonia and acetaldehyde thanks to a specific ethanolamine ammonia lyase [39,64]. The ammonia resulting from this reaction can be used as a cellular supply of reduced nitrogen, and the acetaldehyde is converted to acetyl-CoA that can be used in various bacterial metabolic cycles such as the tricarboxylic acid cycle, the glyoxylate cycle or lipid biosynthesis [64].

The mucus layer of the gut epithelium is composed of mucin proteins that are glycosylated with five major sugars that can be potential nutrients for bacteria: N-acetyl-D-galactosamine, N-acetyl-D-glucosamine, N-acetylneuraminic acid (sialic acid), L-fucose and D-galactose. In cases of gut inflammation, there is an increase in the production and secretion of mucins as a mechanism of maintaining the integrity of the mucus layer. In a study using a mouse model of induced gut inflammation, it has been reported that *E. coli* outgrowth in the inflamed gut consequently leads to an increase in the commensal species expressing sialidases. Such increased activity leads to the release of high amounts of free sialic acid which is efficiently consumed by *E. coli* [65]. In another study, an increase in glycosidase-encoding genes in the microbiome of inflamed gut samples of patients was found to be consistent with an enhanced mucin-degrading activity. Mucin-degrading bacteria such as *Mucispirillum schaedleri*, *Akkermansia muciniphila* and *Bacteroides acidifaciens* expand and mediate the release of less complex sugars (lactose, melibiose, raffinose and galactinol) from mucins [39]. These saccharides and metabolites accumulate and may lead to a decrease in commensal bacteria from the Bacteroidia and Clostridia classes in the inflamed gut, but also confer a growth advantage to *Enterobacteriaceae* as well as to pathogens such as *S. Typhimurium* and *Clostridium difficile* [39,66]. Overall, the composition and abundance of mucosal populations, also known as mucosal biofilm, seems to correlate with health status. Whereas healthy mucosal biofilm is of low microbial density (10^5^–10^6^ cells per mL) and is mostly composed of Firmicutes, Bacteroidetes and some oxygen-tolerant microbes such as lactic acid bacteria and Proteobacteria members, clinical observations from patients with chronic intestinal diseases (IBS, IBD, colorectal cancer) have revealed dense polymicrobial biofilms in the mucus with an increased proportion of *Enterobacteriaceae*. These modified mucosal biofilms have been proposed to be an early warning signal of developing disease and could be considered as the tipping point between a healthy and a diseased state of the gut mucosa [67].

- Diet-derived substrates

It is now well admitted that diet impacts the composition of gut microbiota. For example, a Western-style High-Fat Diet (HFD) is known to induce *Enterobacteriaceae* proliferation in the gut. The enrichment of *Enterobacteriaceae*, largely represented by *Escherichia coli*, has been correlated with impaired glucose homeostasis [68]. In mice receiving an HFD treatment, but not a standard diet, the presence of *E. coli* significantly increased body weight and adiposity and induced impaired glucose tolerance. This work demonstrates the role of commensal *E. coli* in glucose homeostasis and energy metabolism in response to an HFD, indicating contributions of commensal bacteria to the pathogenesis of obesity and type 2 diabetes. An HFD also promotes cardiovascular diseases, in part because of high levels of choline which is converted to trimethylamine (TMA) by gut microbiota. Using a mouse model of diet-induced obesity, Yoo et al. show that chronic exposure to an HFD increases *Escherichia coli* choline catabolism by altering the intestinal epithelial physiology [69]. An HFD impaired the bioenergetics of mitochondria in the colonic epithelium to increase the luminal bioavailability of oxygen and nitrate, thereby intensifying respiration-dependent choline catabolism of *E. coli*. In turn, *E. coli* choline catabolism increased levels of circulating trimethylamine N-oxide, which is a potentially harmful metabolite generated by gut microbiota.

Another example concerns patients suffering from Crohn’s disease (CD), an inflammatory disease of the gut mucosa, associated with a dysbiosis characterized by a high prevalence of a particular type of strain called Adherent and Invasive *Escherichia coli* (AIEC) in the inflamed gut mucosa. These strains possess the ability to adhere to and invade intestinal epithelial cells and to replicate within macrophages [70]. A recent work by Kitamoto et al. shows that the amino acid availability is modified in the inflamed gut and that AIEC reprogram their metabolic pathways to use L-serine in order to gain a fitness advantage over resident microbiota [57]. L-serine is acquired from the diet and can be used as a source of energy after conversion into pyruvate, which is a substrate necessary for glucogenesis and the tricarboxylic acid cycle [71]. While this metabolic pathway has a minor role on AIEC fitness in the healthy gut, this amino acid is a key resource for AIEC expansion during CD intestinal dysbiosis. The described study also successfully demonstrated that restriction of dietary amino acid intake, particularly L-serine, prevents the expansion of *Enterobacteriaceae* in the inflamed gut. Understanding the specific nutrients that *Enterobacteriaceae* use during dysbiosis is essential for developing innovative strategies to restore a balanced gut microbiota.

### 2.3. Consequences of Enterobacteriaceae Proliferation in Disease Progression

**Figure 2:**
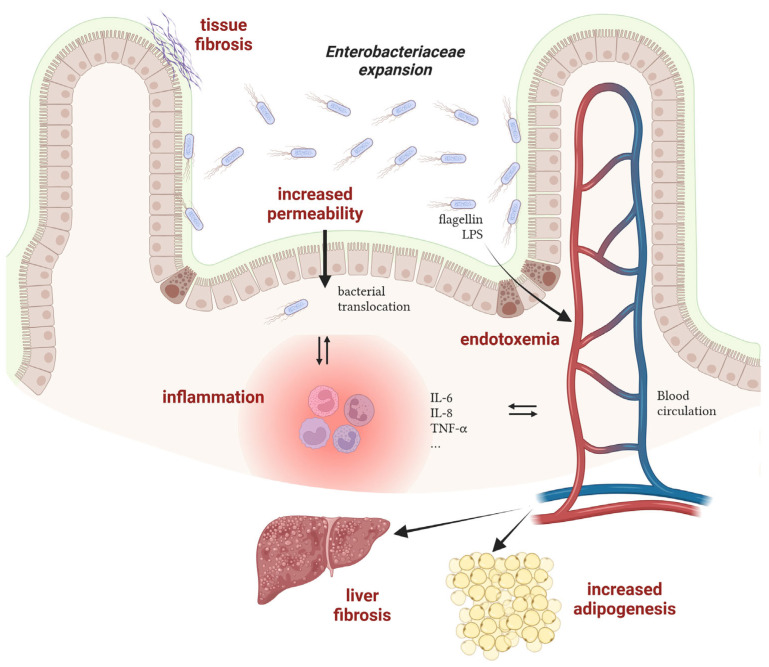
Mechanisms of action of *Enterobacteriaceae* in the progression of inflammatory bowel diseases and obesity-associated diseases. *LPS: lipopolysaccharides*. Created with Biorender.com.

Although the conditions promoting the bloom of *Enterobacteriaceae* have been well investigated these last years, the consequences of such an imbalance on the host are often unknown or underestimated. Nevertheless, works with mechanistic insights into the processes connecting gut dysbiosis and disease are emerging and will allow researchers to unravel whether unbalanced microbial communities participate in disease progression. Hereafter are elements suggesting the harmful role of a bloom of *Enterobacteriaceae* on host health in the case of Crohn’s disease and obesity-associated diseases (Figure 2).

- Crohn’s disease

The relationship between AIEC and intestinal inflammation in CD patients has been extensively investigated in the last 20 years and it is now well admitted that these bacteria participate in the induction and/or maintenance of disease symptoms. Experimental data from CD patients or from AIEC-infected rodents have revealed an increased permeability of the intestinal epithelium in the presence of AIEC [72]. Such dysfunction of the intestinal barrier may cause microbial translocation and trigger an immune response, leading to inflammation. Moreover, adhesion to and invasion of epithelial cells by AIEC also contribute to inflammation, as revealed by increased expression levels of multiple pro-inflammatory effectors such as IL-6, IL-8 and TNF-α [73]. Replication of AIEC within macrophages also leads to TNF-α stimulation [74]. Concomitantly with inflammation, chronic colonization of the intestinal mucosa by AIEC contributes to tissue fibrosis via collagen deposition in the extracellular matrix, potentially leading to bowel obstruction in CD patients [75,76]. Besides AIEC and their contribution to disease, other pathobionts with increased prevalence in CD patients have been identified but their role remains elusive to date [77].

- Obesity-associated diseases

Overweight and obesity, which have tripled worldwide over the last 40 years, are major risk factors for several chronic diseases, including cardiovascular diseases, diabetes, musculoskeletal disorders, nonalcoholic fatty liver disease (NAFLD) and cancers. In most cases, a major gut microbiota alteration is observed in obese patients with a potential impact on the progression of obesity-associated diseases [78]. For instance, a causal link between a strain of *Enterobacter cloacae* (*Enterobacteriaceae* family) and obesity has been deciphered in a work published by the group of L. Zhao [33]. During a clinical study, they found that the gut microbiota of a morbidly obese volunteer was 35% composed of bacteria of the genus *Enterobacter*. After a specific diet program, the weight loss of the patient was concomitant with the disappearance of *Enterobacter* in the gut microbial community. Moreover, colonization of germ-free mice with the *Enterobacter cloacae* strain isolated from the obese human gut leads to the development of obesity in animals fed on a high-fat diet. A second study also demonstrated that *E. cloacae* administration to mice increases subcutaneous fat mass and promotes liver fibrosis, a sign of hepatic damages [79]. In a similar way, supplementation of high-fat diet treated mice with a strain of *E. coli* induces adipogenesis, reduces glucose tolerance and promotes a low-grade systemic inflammation, suggesting a role of commensal bacteria in the pathogenesis of obesity and type 2 diabetes (T2D) [68]. At the molecular level, flagellin, the monomer of the bacterial locomotor appendage, has been shown to induce a pro-inflammatory response in pancreatic beta cells in obese individuals with T2D. Because *Enterobacteriaceae* are more abundant in the gut of individuals with T2D, this flagellin-dependent induction is massive and leads to beta cell dysfunction, a key abnormality leading to hyperglycemia and T2D [80]. In the case of NAFLD, the lipopolysaccharide (LPS) and its endotoxic activity from *E. cloacae*, *Klebsiella pneumoniae* or *E. coli* have been found to participate in liver dysfunction in mice mono-associated with these *Enterobacteriaceae* strains [81]. Altogether, these works reveal that specific bacterial species belonging to the *Enterobacteriaceae* family may causatively contribute to the development of disease in human when their population levels are deregulated in dysbiotic conditions.

## 3. Conclusions

Taxonomic analyses of the gut microbiota from healthy individuals or patients suffering from various intestinal or extra-intestinal diseases reveal that gut dysbiosis in patients are often characterized by an expansion of Proteobacteria, particularly *Enterobacteriaceae*, making this trait a potential marker to detect the onset of disease. Recent studies also decipher some conditions or parameters associated with dysbiosis that could explain *Enterobacteriaceae* proliferation in the gut and how such an imbalance could affect host health and disease progression. A better understanding of the mechanisms governing disease state and gut dysbiosis, especially *Enterobacteriaceae* bloom, would improve the development of new approaches for the manipulation of gut bacterial communities in order to sustain or restore homeostasis and improve host health.

## Figures and Tables

**Table 1:** Studies reporting nutrient sources available for *Enterobacteriaceae* during intestinal dysbiosis.

Nutrient	Bacterial Species	References
Ethanolamine	*S. Typhimurium* ^1^ *C. rodentium* ^2^	[51] [52]
Lactate	*S. Typhimurium*	[53]
Glucarate/galactarate	*S. Typhimurium* Commensal *E. coli*	[54]
1,2-propanediol	*S. Typhimurium*	[55]
Succinate	*S. Typhimurium*	[56]
L-Serine	Adherent-invasive *E. coli* *C. rodentium*	[57] [58]
Glycerol	Commensal *E. coli*	[59]
Carnitine and Acylcarnitines	*E. coli*	[60]

^1^ *Salmonella enterica* serovar *Typhimurium*. ^2^ *Citrobacter rodentium*.

## Data Availability

Not applicable.
